# Omega-3 fatty acids as treatments for mental illness: which disorder and which fatty acid?

**DOI:** 10.1186/1476-511X-6-21

**Published:** 2007-09-18

**Authors:** Brian M Ross, Jennifer Seguin, Lee E Sieswerda

**Affiliations:** 1 Northern Ontario School of Medicine, Public Health Program, and Departments of Chemistry and Biology, Lakehead University, Thunder Bay, Ontario, Canada; 2 Department of Psychology, Thunder Bay, Ontario, Canada; 3 Thunder Bay District Health Unit and Northern Ontario School of Medicine, Lakehead University, Thunder Bay, Ontario, Canada

## Abstract

**Background:**

A growing number of observational and epidemiological studies have suggested that mental illness, in particular mood disorders, is associated with reduced dietary intake and/or cellular abundance of omega-3 polyunsaturated fatty acids (PUFA). This has prompted researchers to test the efficacy of omega-3 PUFA in a range of different psychiatric disorders. We have critically reviewed the double blind placebo controlled clinical trials published prior to April 2007 to determine whether omega-3 PUFA are likely to be efficacious in these disorders.

**Results:**

Most trials involved a small number of participants but were largely well designed.  Omega-3 PUFA were well tolerated by both children and adults with mild gastrointestinal effects being the only consistently reported adverse event. For schizophrenia and borderline personality disorder we found little evidence of a robust clinically relevant effect. In the case of attention deficit hyperactivity disorder and related disorders, most trials showed at most small benefits over placebo. A limited meta-analysis of these trials suggested that benefits of omega-3 PUFA supplementation may be greater in a classroom setting than at home. Some evidence indicates that omega-3 PUFA may reduce symptoms of anxiety although the data is preliminary and inconclusive. The most convincing evidence for beneficial effects of omega-3 PUFA is to be found in mood disorders. A meta-analysis of trials involving patients with major depressive disorder and bipolar disorder provided evidence that omega-3 PUFA supplementation reduces symptoms of depression. Furthermore, meta-regression analysis suggests that supplementation with eicosapentaenoic acid may be more beneficial in mood disorders than with docosahexaenoic acid, although several confounding factors prevented a definitive conclusion being made regarding which species of omega-3 PUFA is most beneficial. The mechanisms underlying the apparent efficacy of omega-3 PUFA in mood disorders compared to schizophrenia are discussed as is a rational for the possibly greater efficacy of EPA compared to DHA.

**Conclusion:**

While it is not currently possible to recommend omega-3 PUFA as either a mono- or adjunctive-therapy in any mental illness, the available evidence is strong enough to justify continued study, especially with regard to attentional, anxiety and mood disorders.

## Background

Omega-3 fatty acids are a type of polyunsaturated fatty acid (PUFA). PUFA are so-called because they are not 'saturated' with hydrogen atoms at multiple (poly) locations within the molecule and, as a result, contain two or more carbon-carbon double bonds. They form one of the three main classes of fatty acids, the others being saturated, in which all available hydrogen atom positions are filled, and monounsaturated, in which a single carbon-carbon double bond exists. PUFA are subdivided into the omega-3 (n-3) series (the first double bond is 3 carbons from the end (omega) carbon atom of the molecule) that are synthetically derived from linoleic acid (LA), and the omega-6 (n-6) series which are derived from alpha-linolenic acid (ALA), both 18 carbon atom containing fatty acids. LA and ALA are termed essential fatty acids because mammalian cells are unable to synthesize these fatty acids from simpler precursors. LA can be converted sequentially via a biosynthetic pathway into other omega-6 fatty acids, the 18 carbon gamma linolenic acid (GLA), and the 20 carbon arachidonic (AA) and dihomogammalinolenic acids (DGLA). Similarly, ALA is converted into longer chain omega-3 fatty acids such as 20 carbon eicosapentaeoic acid (EPA) and 22 carbon docosahexaenoic acid (DHA). Increasing evidence indicates, however, that although LA and ALA *can *be converted into their longer chain length metabolites, the rate of conversion in humans is very slow, resulting in an estimated 2 to 10% of ALA being converted to DHA or EPA [1,2]. This suggests that a major source of the longer chain polyunsaturated fatty acid species such as EPA and DHA is likely to be dietary. Such a view is supported by data that supplementation with fish oils can markedly elevate the cellular levels of both these omega-3 PUFA [3].

Omega-3 PUFA are of particular interest from a nutritional standpoint since the intake of these fatty acid is considered to be low in Western diets [4]. They have long been investigated for their cardioprotective and anti-inflammatory roles, which has lead to their increased use as dietary supplements [5]. A new application for omega-3 fatty acids has emerged recently, the treatment of certain forms of mental illness. Such a use is biologically plausible given that omega-3 fatty acids, in particular DHA, are abundant in the brain and are involved in, or modulate, the mechanism by which brain neurons communicate [6]. They have been shown to alter the functioning of neural systems utilising dopamine and serotonin, both of which are thought to play an important role in mental illness and are major targets of psychoactive medications [6,7]. Furthermore, animal models of mental illness have suggested that omega-3 fatty acids can affect brain processes such as those that control mood and anxiety [8].

## Methods

This review considers the findings of the clinical trials that form the basis of these developments and whether or not they offer convincing support for the use of omega-3 PUFA as a treatment for mental illness. To accomplish this we utilized PubMed and PsycINFO to identify placebo-controlled clinical trials of omega-3 fatty acids in psychiatric disorders. The strategy employed was to search for articles which contained search terms from list 1 (schizophrenia, psychosis, delusion, bipolar, mania, depression, mood, personality, anxiety, obsession, attention, hyperactivity, autism, retardation, somatoform, anorexia, bulimia, cognitive) and from list 2 (omega-3, n-3 alpha linoleic, eicosapentaenoic, docosahexaenoic, EPA, DHA, ALA). We also searched using Pubmed for omega-3, n-3, alpha linoleic, eicosapentaenoic, docosahexaenoic, EPA, DHA, or ALA limited by clinical trial. We reviewed these trials with respect to their findings, statistical analysis and overall trial quality.

For all trials we assessed the evidence that trial participants who had received omega-3 PUFA had improved psychopathology compared to those who received placebo treatment. This was accomplished by extracting or calculating the change-from-baseline scores for the appropriate clinical rating scale for each disorder, and then calculating the mean difference in change-from-baseline scores between the placebo and omega-3 PUFA groups. A standardized or unstandardized difference value of zero indicates that there was no difference in the effect upon psychopathology between the two groups. For the attention deficit hyperactivity disorder (ADHD) and depression trials we constructed Forest plots of the standardized mean differences in change-from-baseline scores for the comparable trials. For the depression trials, we calculated an inverse-variance-weighted overall estimate of effect using the random effects method [9] as well as conducting a meta-regression to identify sources of heterogeneity between the trials [9]. We conducted all analyses using STATA version 9.2 (Stata Corp, College Station, TX).

The quality of the trial design was assessed using the Jadad scoring system [10] which takes into account whether the trial was double blind, randomized using an appropriate method, whether withdrawals from the trial were described and whether an appropriate placebo was used. A higher score indicates a higher apparent design quality. The trials considered varied in quality, utilised a variety of clinical outcome measures, and used a variety of fatty acid formulations including DHA or EPA rich fish oils, as well as a semi-purified (96%) ethyl-EPA ester. A further aim of this review was therefore to assess the evidence of whether EPA or DHA is more efficacious in any form on mental illness. Notably, all trials utilised the long chain (20 carbon atoms or greater) omega-3 PUFA rather than the 18 carbon atom ALA.

## Results

The effect of omega-3 PUFA upon psychopathology was investigated in a range of psychiatric disorders that we will consider by general disease class.

### Attention deficit hyperactivity disorder and related disorders

Attention deficit/hyperactivity disorder (ADHD) is a common disorder of school age children (although adults can also be affected). Estimates vary but the prevalence of ADHD is thought to be in the range of 2 – 7% of children [11]. As the name suggest ADHD comprises hyperactivity and a range of attentional deficits including problems listening, paying attention and finishing tasks, as well as interrupting others [12]. The illness is also associated with social withdrawal, shyness and anxiety and around one quarter of children with ADHD may also show learning difficulties including problems with reading and writing [13]. Such behavioural problems can affect academic attainment as well as relationships with family and peers.

Over 80% of children with ADHD in the US are treated with stimulants such as methylphenidate (Ritalin). While efficacious in some patients, these medications have significant side effects including decreased appetite, insomnia, impaired growth and irritability [13]. Although the aetiology of ADHD is unclear, one suggested contributory factor is diet including the abnormal intake and abundance of various types of PUFA [14,15]. Reduced PUFA levels have been reported in both plasma and erythrocyte membranes of children with ADHD [16-19], while a preliminary study suggests that omega-3 fatty acid breakdown occurring consequent to oxidative stress is increased [20]. Such findings have led to the hypothesis that lack of sufficient amounts of specific fatty acids affects brain function in such a way as to cause or worsen the symptoms of ADHD [14]. Early studies examining the effects of essential fatty acid supplementation focused exclusively on omega-6 fatty acids. In double-blind placebo-controlled trials of mixtures of gamma linoleic acid and LA in children with ADHD both Aman and colleagues [21] and Arnold and colleagues [22] reported no significant improvement in symptoms. Despite such negative results, the encouraging data emanating from early trials of omega-3 fatty acids in depression and schizophrenia (described later in this review) prompted investigation of these fatty acids in ADHD and related disorders.

Interest in the interaction between diet and attentional disorders is currently high with Joshi and colleagues [23] reporting decreased hyperactivity in children with ADHD receiving flax oil (an oil rich in the 18 carbon omega-3 fatty acid ALA) in a non-placebo-controlled trial. Furthermore, Fontani and colleagues [24] reporting that omega-3 fatty acid supplementation enhances measures of attention in healthy adults. In addition, five double-blind placebo-controlled trials have been carried out that examine how omega-3 PUFA supplementation affects attention and hyperactivity (Table 1). The trials used a variety of outcome measures. We have summarized a small subset of the results with a focus on two aspects of ADHD, attention and hyperactivity (Table 1). The first ADHD study was conducted by Voigt and colleagues [25]. These researchers tested the effect of 345 mg/day DHA for 4 months upon 63 children with the disorder. Although blood DHA levels were increased in the active treatment group, no significant difference in symptoms was observed compared to placebo and assessed using the Child Behaviour Checklist [26] and the Conners' rating scale [27] (Conner's scale data is not shown in the report). No significant benefit of a DHA/EPA mixture compared to placebo was also reported by Hirayama and colleagues [28]. Rather than being in capsule form, the DHA supplementation was administered through DHA fortification of food stuffs. As such, the daily dose can only be estimated but was approximately 100 mg EPA and 500 mg DHA/day. The authors report that the number of different symptoms present which comprise an ADHD diagnosis (as described in the Diagnostic and Statistical Manual for Mental Disorders Version IV abbreviated to DSM-IV [12]), was not changed compared to placebo by omega-3 PUFA administration. It is important to note, however, that the authors did not assess the severity of symptoms, merely their presence. Such a measure may not possess sufficient sensitivity to detect smaller improvements in the condition however.

**Table 1:** Double blind placebo-controlled trials of omega-3 fatty acids upon attention and hyperactivity in disorders diagnosed in childhood.

***Ref***	***Jadad score***	***Disorder***	***Meds***	***Age***	***Gen***	***Omega-3 fatty acid***	***Prep type***	***Duration***	***N***	***Outcomemeasure***	***Average baseline score***	**Δ**	***Statistical significance vs. placebo***
25	4	ADHD	100%	9	22%	345 mg/day EPA	Crude Mixture	4 months	54	CBC-Attention	66	+0.4	No
28	5	ADHD	15%	9	20%	100 mg/day EPA 512 mg/day DHA	Crude mixture in food	2 months	40	#DSM-Attention	9.5	-1	No
										#DSM-Hyperactivity	2	0	No
29	5	LD	NS	10	20%	186 mg/day EPA 480 mg/day DHA^2^	Crude mixture	12 weeks	29	CPRS DSM-Attention	63	-4.6	Yes
										CPRS DSM-Hyperactivity	67	-1.0	No
30	3	'ADHD-like'	79%	10	12%	80 mg/day EPA 480 mg/day DHA^1^	Crude mixture	4 months	47	PDBDS-Attention	22	+0.2	No
										PDBDS-Hyperactivity	22	-0.6	No
32	5	DCD	0%	9	33%	558 mg/day EPA 174 mg/day DHA^3^	Crude mixture	12 weeks	117	CPRS DSM-Attention	65	-3.5	Yes^4^
										CPRS DSM-Hyperactivity	61	-3.8	Yes^4^
										Spelling age (months)	94	+5.4	Yes
										Reading age (months)	97	+6.2	Yes
34	4	Autism	0%	10	18%	840 mg/day EPA 700 mg/day DHA	Crude mixture	6 weeks	13	ABC-Hyperactivity	29	-7.0	No

ADHD, LD and DCD are Attention Deficit Hyperactivity Disorder, Learning Difficulties and Developmental Coordination Disorder respectively. Δ refers to the difference between the change from baseline values for placebo and treatment groups (change from baseline symptom scores for the omega-3 fatty acid group minus change from baseline scores for the placebo group). Trial quality was assessed using Jadad design quality scores which can range from 0 to 5 with 5 being best. Outcome measures are CBC – Attention: Child Behaviour Checklist – Attention Subscale; PDBDS – Attention/Hyperactivity: Attention or Hyperactivity Subscale of Parents Disruptive Behaviour Disorder Scale, #DSM – Attention/Hyperactivity: Number of DSM ADHD Attention or Hyperactivity symptoms present, CPRS DSM – Attention/Hyperactivity: DSM Attention/Hyperactivity Subscale of Conner's Parent Rating Scale Clinical Global Impression, ABC-hyperactivity: Aberrant Behaviours Checklist [34] For reading and spelling age a higher age is better hence positive Δ indicates improvement over placebo, for all other scales 0 is best hence negative Δ indicates improvement over placebo. The average baseline score is the average of the omega-3 and placebo group scores at the start of the trial. Age refers to the average age in years of the active and placebo groups. Gen (gender) refers to the % of female participants in the trial. Meds refers to the percentage of trial participants who were in receipt of conventional medications for attentional deficit or hyperactivity. Prep. type describes whether a crude oil mixture or a purified fatty acid was used. Notes: ^1–3 ^Formulation also delivered omega-6 fatty acids: ^1 ^40 mg/day arachidonic acid and 96 mg/day gamma linoleic acid, ^2 ^96 mg/day gamma linoleic acid, 864 mg/day linoleic acid and 42 mg/day arachidonic acid, ^3 ^60 mg/day linoleic acid. ^4 ^According to the report it is unclear as to whether statistical significance was calculated using raw scores rather than transformed t-scores. Using the data in the paper the statistical significance of the t-score comparison cannot be calculated.

The three other trials differed from the others in that they did not utilize children with formally diagnosed ADHD but rather ADHD-related symptoms, as determined using one or more rating scales. As such the subject groups are likely to be more heterogeneous than found in the trials conducted by Voigt and colleagues [25] and by Hirayama and colleagues [28], and likely contain participants who would not meet the DSM-IV criteria for clinical ADHD. Richardson and Puri [29] tested the effects of a supplement containing a mixture of omega-3 and omega-6 fatty acids in children with learning difficulties (mainly dyslexia) who also exhibited ADHD-like features assessed using the Conner's Teacher Rating Scale. Since the Conner's scale scores are influenced both age and gender, scores are routinely transformed using standard population values which convert the raw scores into standardised t-scores with mean = 50 and standard deviation of 10. A score greater than two standard deviations from the mean (> 70) correlates well with a clinical diagnosis of ADHD [27]. It is notable that the participants in the Richardson and Puri trial [29] have a mean t-score of less than 70, indicating that most of the participants likely would not meet the criteria for clinical ADHD. In the trial, subjects were treated with placebo or 186 mg EPA, 480 mg DHA, 96 mg GLA, 864 mg LA and 42 mg AA/day for 12 weeks. It is unclear why the authors chose to include the omega-6 PUFA GLA in the formulation given earlier negative findings [21,22], however, as noted below, the inclusion of AA and GLA, a precursor of AA, may be beneficial. The authors report statistically significant improvements upon a range of ADHD-like symptoms assessed using the Conner's scale including psychosomatic symptoms and inattention, with cognitive problems and anxiety approaching statistical significance. Examination of the difference between placebo and omega-3 PUFA groups indicates, however, that the beneficial effect of the omega-3 PUFA treatment was, at most, modest (Table 1). The small number of study participants in this trial is also a significant weakness. A comparable study conducted by Stevens and colleagues [30] examined fatty acid supplementation in children with ADHD-related symptoms, such as inattention, hyperactivity, and other disruptive behaviours. Subjects received either placebo or 480 mg DHA, 80 mg EPA, 40 mg AA and 96 mg GLA/day for 4 months. No statistically significant difference between placebo and active groups was observed for any rating scale when those who completed the trial were compared (Table 1). Using an intent-to-treat analysis did however indicate a significant benefit of fatty acids upon attention assessed by teachers, and conduct assessed by parents using the Disruptive Behaviours Scale [31]. An intent-to-treat analysis is considered more valid since it includes participants who withdrew from the trial (who are likely to be more be severely ill) by carrying forward their baseline score (intake score) to the end of the trial even though they have not participated fully, or at all, in the study treatment protocol. Notably, the authors also report that by the end of the trial a significantly greater proportion of children had moved out of the clinically significant range for oppositional/defiant behaviour than in the treatment group. While these improvements are interesting, the magnitude of the effects described are relatively small and the trial, like that by Richardson and Puri [29], is at best suggestive of a small effect upon attentional deficit and/or hyperactivity.

A further trial [32] used a substantially larger sample size to investigate the effects of mixed omega-3 and omega-6 supplementation in 117 children with Developmental Coordination Disorder (DCD) [33]. DCD is characterised by specific impairments of motor function and shows substantial overlap with ADHD in terms of difficulties with organizational skills and attention. Similar to the author's earlier study [29], the Conner's rating scale values at entry into are indicative of a study population in which the majority of participants would likely not meet the criteria for ADHD (quoted in the report as 69% of participants). Using a double blind, placebo controlled high quality design (Jadad score = 5) children received either 558 mg EPA, 174 mg DHA and 60 mg GLA/day or placebo for 12 weeks. The fatty acid supplementation had no significant benefit over placebo upon motor function, but, notably, resulted in statistically significant improvements in reading age, spelling age and on all except one Conner's'subscale (opposition, cognitive problems, hyperactivity, anxiety, perfectionism, social problems, impulsivity, and inattention). A concern arises, however, from the fact that while the p-values for the difference between omega-3 PUFA and placebo groups reported by the authors appear impressive, the actual magnitude of the difference between the groups is much less so. For example, the change in normalised t-scores for DSM-IV Attention scales in the PUFA group was better than the placebo group by only 3.8 points (or 38% of 1 standard deviation) while the quoted statistical significance is *p *< 0.00001. Information provided in the report suggests two possible explanations. Firstly, the authors state [32] that they used non-parametric tests which, depending on how the data ranks in each group, could produce such an outcome although this is unlikely to occur. Secondly, they state [32] that "group comparisons were therefore performed by using the raw scores obtained by summing across all items." This suggests that the statistical significance quoted is for comparison of raw scores, rather than the t-scores presented in the paper. Whatever the explanation, it appears that the magnitude of difference between placebo and PUFA groups is not as striking as is suggested by the very small p-values. Furthermore, since most of the participants (69%) did not have clinically significant inattention and/or hyperactivity, it is unclear what the clinical relevance of the authors observations are, if any. On the other hand, the authors did find an effect and it will be of great interest to utilise the same formulation in children who have clinically significant symptoms as defined using either the Conner's scale or by psychiatric assessment.

The most recent trial [34] assessing the effect of omega-3 PUFA upon hyperactivity (but not inattention) involved a group of children with autism. Autism is chiefly characterised by limited behaviour patterns, reduced social interaction and delayed language, however hyperactivity is also frequently observed [12]. Whether there is an aetiological overlap between hyperactivity in ADHD and in autism is unclear. The authors administered a supplement delivery a daily dose of 800 mg of EPA and 700 mg of DHA or a coconut oil placebo for 6 weeks (a considerably shorter duration than most trials), and assessed psychopathology using the Aberrant Behaviours Checklist [35]. Omega-3 PUFA showed no statistically significant benefit over placebo on any of the ABC scales including irritability, social withdrawal, stereotypy, inappropriate speech or hyperactivity. The authors highlight the fact that hyperactivity showed the greatest difference relative to the placebo group although much of the inter-group difference was due to a worsening of hyperactivity during the trial in the placebo group. The small size and short duration of the trial are problematical and further trials will be required to confirm or reject a previous observation obtained using and open-label trial that omega-3 PUFA supplementation is beneficial in autism [36].

**Figure 1:**
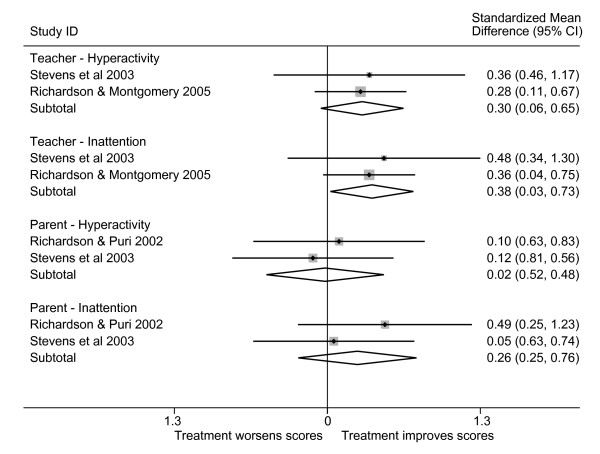
*Standardized mean differences in ADHD-related symptoms in omega-3 PUFA-treated compared to placebo-treated subjects*. SMD are the standardized mean differences (scaled to standard deviations) in the change-from-baseline scores in the placebo and treatment groups, along with 95% confidence intervals. A summary of each study's characteristics can be found in Table 1. Because of the incomparability of the instruments used in several of the studies, we were only able to include three studies in this summary. We have shown separate estimates for hyperactivity and inattention subscales, as well as distinguished parent and teacher ratings. There are not yet sufficient sufficiently comparable data to justify calculating an overall summary. We have, however, provided preliminary inverse-variance-weighted averages of the two studies in each subsection.

The variety of disorders and outcome measures used in the ADHD-related trials made direct comparison amongst them difficult. Three trials [29,30,32] utilised comparable measures of hyperactivity and inattention, however, which allowed us to provide preliminary estimates of the effect of omega-3 fatty acid supplementation on inattention and hyperactivity as assessed by parents and teachers (Figure 1). These estimates are exploratory since the grouping into separate parent-teacher and inattention-hyperactivity categories was based on the available data, not on any *a priori *hypothesis. Furthermore, there was not sufficient comparable data to attempt to calculate an overall estimate of effect. From these limited data it appears that teachers may tend to report a greater beneficial effect than do parents, and that there may be a greater effect on inattention than on hyperactivity.

In summary, omega-3 containing PUFA supplements did not perform better than placebo in children with clinically diagnosed ADHD, but may have had a modest effect on attention and hyperactivity symptoms in children with DCD. The data are, however, inconclusive at this time. A trial of a similarly formulated supplement to that used in the DCD trial [32] in clinically defined ADHD has not yet been published. Such an experiment is needed to resolve the question of whether omega-3 PUFA supplementation is beneficial in ADHD, as are trials which take place over longer time intervals. Finally, because the trials used a variety of PUFA formulations, no conclusion can currently be drawn regarding which omega-3 PUFA, if any, is most effective.

### Anxiety disorders

The investigation of the efficacy of omega-3 PUFA in anxiety disorders is a field in its infancy. Anxiety disorders comprise a variety of disorders characterised by abnormal fears and phobias such as panic disorder, generalised anxiety disorder, obsessive compulsive disorder and social phobia [12]. To date only 2 placebo controlled trials have been performed (Table 2). Omega-3 fatty acid supplementation has been assessed using double blind, placebo controlled trial in obsessive compulsive disorder (OCD), as well as anxiety associated with substance use. Using a crossover design Fux and colleagues [37] administered placebo or 2 g/day ethyl EPA for 6 weeks followed by a liquid paraffin placebo or EPA for a further 6 weeks to 11 patients with OCD. Symptoms were assessed using the Yale-Brown Obsessive Compulsive Scale [38], the Hamilton Depression Rating Scale (HDRS [39]) and the Hamilton Anxiety Rating Scale [40]. The authors found no significant difference compared to placebo treatment on any of the scales and concluded that omega-3 fatty acids were not efficacious (it is worth noting that depression and anxiety symptoms in the patient groups were mild). The study, however, utilized a very small sample size and administered fatty acids for a much shorter time period than used in the most other trials. As such, although this trial reports a negative finding, its limitations leave open the question of whether omega-3 PUFA have any efficacy in OCD. A second recent trial has generated more positive results although the findings should be considered as very preliminary. In a double-blinded randomised trial Buydens-Branchley and Branchley [41] administered either a vegetable oil placebo or an omega-3 supplement providing 2.2 g EPA and 0.5 g DHA/day to 24 men being treated for substance use. Using a self-administered test scale which assessed anger, tension and anxiety, and mood (Profiles of Mood states scale [42]) the authors report a significantly better improvement in the tension and anxiety subscale compared to placebo. The use of patients who do not have a clinically defined anxiety disorder is a limiting factor of the trial, as is the small size and use of a self-rating scale. Thus, while these trials are not conclusive with regard to the potential efficacy of omega-3 PUFA supplementation, they do provide a rationale for conducting further trials in populations with clinically well defined anxiety disorders. Interestingly, anxiety was also reduced in an open-label trial (i.e. a non-blinded trial having with no placebo) of an omega-3/omega-6 PUFA mixture is students writing their examinations [43] whereas supplementation with 443 mg EPA and 319 mg/day DHA for 2 months reduced plasma catecholamine levels (adrenaline, noradrenaline and dopamine) relative to those receiving a soybean lecithin placebo [44]. Such an observation increases the biological plausibility of omega-3 PUFA reducing the symptoms of anxiety given the central role that the hypothalamic-pituitary-adrenal axis (the major control mechanism for catecholamine synthesis) in these disorders [6]. It is also of note that reduced omega-3 PUFA abundance has recently been reported in non-depressed patients with social phobia, another form of anxiety disorder, opening up a further avenue for clinical investigation [45].

**Table 2:** Double blind placebo-controlled trials of omega-3 fatty acids upon in anxiety disorders.

*Ref*	*Jadad score*	*Disorder*	*Meds*	*Age*	*Gen*	*Omega-3 fatty acid*	*Prep type*	*Duration*	*N*	*Outcome measure*	*Average baseline score*	Δ	*Statistical significance vs. placebo*
37	4	OCD	100%	34	73%	2 g/day EPA	Ethyl EPA	6 weeks	11	YBOCS	26	-0.9	No
										HDRS	11	NS	No
										HARS	14	NS	No
41	4	SUA	42%	51	0%	450 mg/day EPA 100 mg/day DHA	Crude mixture	3 months	24	POMS-T	4.3	-2.9	Yes

OCD and SUA are obsessive compulsive disorder and substance use associated anxiety respectively. Δ refers to the change from baseline symptom scores for the omega-3 fatty acid group minus change from baseline scores for the placebo group. Jadad design quality scores can range from 0 to 5 with 5 being best. The symptom scales used are the Yale-Brown Obsessive Compulsive Scale (YBOCS), Hamilton Depression Rating Scale (HDRS), Hamilton Anxiety Rating Scale (HARS) and the tension subscale of the Profile of Mood Scale (POMS-T). Zero is best for all scales hence a negative Δ value indicates an improvement over placebo. The average baseline score is the average of the omega-3 and placebo group scores at the start of the trial. Gen (gender) refers to the % of female participants in the trial. Meds refers to the percentage of trial participant who were in receipt of conventional medications for anxiety disorders. Prep. type describes whether a crude oil mixture or a purified fatty acid was used. NS – not stated.

### Schizophrenia

Fatty acids have also been the focus of intense study in the psychotic disorder schizophrenia [12]. Schizophrenia is a severe mental illness with a prevalence of 1 – 2% [46] that, like depression, has a complex and poorly understood aetiology. The disease is episodic and is characterised by two classes of symptoms: positive, which include hallucinations, delusions, disorganized thought and behaviour, and negative, such as flattened mood, poverty of speech, and deficits in goal directed behaviours which account for most of the morbidity of the illness [47]. The disorder is treated with a variety of anti-psychotic medications all of which have limited efficacy (treatment of the debilitating negative symptoms is poor) and significant side effects including motor function, obesity and, more controversially, diabetes [6]. More effective treatments are needed for schizophrenia and a variety of different approaches have been tried including one that postulates that schizophrenia is associated with an abnormality in the cell membrane, termed the phospholipid hypothesis [47]. A large number of studies have examined fatty acid abundance in the disorder [48] including reports of decreased PUFA abundance of a variety of body tissues [49]. The PUFA affected vary between studies leading to the recent proposal that fatty acid alterations in the disorder are secondary to altered dietary intake occurring as a consequence of the disease process [48].

Despite the inconsistent nature of the fatty acid abundance data, a number of clinical trials utilising fatty acid supplementation in schizophrenia defined according to DSM criteria have been carried out. The earliest trials utilized n-6 fatty acids, mainly gamma linoleic acid. The outcome of these small studies was largely negative [49]. More recently, the efficacy of omega-3 fatty acids was examined in a series of double blind trials. All the trials utilised the Positive and Negative Syndrome Scale (PANSS) [50] to monitor disease severity in patients with similar baseline psychopathology (Table 3). Using a double-blind placebo-controlled design, Peet and colleagues [3] compared the effect of administering an EPA-enriched oil containing 2 g/day EPA, a DHA-enriched oil containing 2 g/day DHA or a corn-oil placebo for 3 months upon schizophrenic symptom onset in 45 patients who were in receipt of conventional anti-psychotic medications. The authors reported that at the end of the trial the PANSS score in the EPA groups was significantly lower than in the placebo group although the difference was not large (Table 3). A comparison of change from baseline values for omega-3 and placebo groups was not performed. In addition, a significantly greater proportion of participants in the EPA group had PANSS scores that improved by over 25% compared to that in the placebo and DHA groups. This success criterion was not an *a priori *outcome measure, however, and, in any case, the clinical significance of a 25% improvement in symptoms is unclear [51]. The authors also conducted a second double-blind placebo-controlled trial in which 26 unmedicated patients with schizophrenia were treated with an EPA-enriched oil providing an equivalent of 2 g/day EPA for 3 months [3]. The trial was unusual in that patients received conventional antipsychotic medications if they required them during the EPA treatment period, and as such the level and type of medication of subjects tended to vary during the trial, an important confound which hinders interpretation of the findings. At the end of the trial the EPA treated group had significantly lower PANSS scores than the corn-oil placebo group, although again a change from baseline comparison was not performed. Moreover, the difference between active and placebo groups was not large (Table 3). The authors also report significantly fewer days on antipsychotic treatment during the trial in the EPA group and that at the end of the trial 4 out of 15 EPA-treated subjects had not received any additional medication throughout the trial, while all placebo-treated patients were medicated with conventional drugs. While an interesting finding, the short duration of the trial combined with the episodic nature of the disorder makes it difficult to assess the clinical relevance of such an observation.

**Table 3:** Double-blind placebo controlled trials of omega-3 fatty acids in schizophrenia.

*Ref*	*Jadad score*	*Meds*	*Age*	*Gender*	*Omega-3 fatty acid*	*Prep. type*	*Duration*	*N*	*Average baseline score*	Δ	*Statistical significance vs. placebo*
3	5	100%	43	38%	2 g/day EPA	Crude mixture	12 weeks	29	75	-4.1	Yes
			43	29%	2 g/day DHA	Crude mixture	12 weeks	40	73	+2.2	No
3	5	0%^1^	35	40%	2 g/day EPA	Crude mixture	12 weeks	26	75	-3.6	Yes
52	5	100% (typical)	39	54%	1 g/day EPA	Ethyl ester	12 weeks	21	74	+7.0	No
			37	47%	2 g/day EPA	Ethyl ester	12 weeks	17	75	+4.2	No
			38	57%	4 g/day EPA	Ethyl ester	12 weeks	22	77	+2.5	No
		100% (atypical)	39	54%	1 g/day EPA	Ethyl ester	12 weeks	23	76	+5.2	No
			37	47%	2 g/day EPA	Ethyl ester	12 weeks	26	82	-0.9	No
			38	57%	4 g/day EPA	Ethyl ester	12 weeks	23	81	+10.6	No
		100% (clozapine)	39	54%	1 g/day EPA	Ethyl ester	12 weeks	16	79	-10.1	No
			37	47%	2 g/day EPA	Ethyl ester	12 weeks	16	81	-17.8	Yes
			38	57%	4 g/day EPA	Ethyl ester	12 weeks	13	74	-6.9	No
		100% (all meds)	39	54%	1 g/day EPA	Ethyl ester	12 weeks	60	77	-1.0^2^	No^2^
			37	47%	2 g/day EPA	Ethyl ester	12 weeks	59	78	+5.4^2^	No^2^
			38	57%	4 g/day EPA	Ethyl ester	12 weeks	58	77	-3.7^2^	No^2^
53	4	100%	40	39%	3 g/day EPA	Ethyl ester	12 weeks	87	75	+2.0	No
							16 weeks	87		+1.0	No
56	3	100%	45	NS	3 g/day EPA	Ethyl ester	9 weeks	40	75	-9.5	Yes
							12 weeks	40		-7.3^3^	Yes

Δ is refers to the change from baseline symptom scores for the omega-3 fatty acid group minus change from baseline scores for the placebo group. Jadad design quality scores can range from 0 to 5 with 5 being best. The symptom scale used in all the trials is the Positive and Negative Symptom Scale (PANSS). Zero is best for this scale hence a negative Δ value indicates an improvement over placebo. The average baseline score is the average of the omega-3 and placebo group scores at the start of the trial. Gen (gender) refers to the % of female participants in the trial. Meds refers to the percentage of trial participant who were in receipt of conventional medications for schizophrenia. Prep. type describes whether a crude oil mixture or a purified fatty acid was used. Notes: ^1^Although trial participants were not in receipt of medication at commencement, they were giving anit-psychotic medications as required during the trial. ^2^The primary outcome measure was stated by the authors to be 12 weeks, but this was not reported in the text; therefore this estimate is an approximation based on Figure 1 in Emsley and colleagues [56]. ^3 ^Calculated from Tables 6 – 8 in Peet and Horrobin [52].

Peet and Horrobin [52] followed up these trials with a dose-ranging placebo-controlled double-blind study of 1, 2, or 4 g/day ethyl-EPA or a liquid paraffin placebo for 12 weeks in a group of 115 medicated patients with schizophrenia. They report that 2 g/day EPA (but not 1 or 4 g/day) improved change-from-baseline scores in patients treated with the atypical antipsychotic clozapine, but not in those treated with typical or non-clozapine atypical antipsychotics [52]. Indeed, in those treated with typical and non-clozapine atypical neuroleptics all doses of EPA except one actually performed less well than placebo (mean difference scores were positive). A clozapine-treatment specific effect of EPA in schizophrenia seems unlikely and instead may be due to chance alone. The authors do not state the effect of EPA when all the medication groups are combined. Using the data presented in the paper the change-from-baseline values for EPA and placebo groups can be calculated (Table 3). Assuming a normal distribution of the data it is also possible to calculate 95% confidence intervals for the difference between placebo EPA-treated groups these being for 1 g/day EPA: -4.7 to + 6.7; 2 g/day EPA: -12.7 to +1.8; 4 g/day EPA: -2.7 to +10.2. Since all of these intervals contain the value zero i.e. no difference between active and placebo groups, it is likely that none of the EPA doses significantly improved symptoms compared to placebo when subjects are analysed as a whole.

Besides the researchers lead by Peet, two other groups have investigated the use of EPA in schizophrenia using double-blind placebo controlled trials. Fenton and colleagues [53] administered either 3 g/day ethyl-EPA or a mineral oil placebo to 87 medicated patients with schizophrenia for 16 weeks. They observed no significant difference in PANSS scores between the EPA and placebo groups at any of the time points reported (1, 2, 4, 8, 12 or 16 weeks). It has been argued that erythrocyte EPA levels rose significantly in the placebo group, suggesting that the informed consent procedure lead to participants consuming omega-3 fatty acid rich foods or taking fish oils supplements thereby defeating the placebo element [54]. It is unlikely that such an explanation explains the lack of effect given that the change from baseline symptom scores in both placebo and omega-3 groups were low compared to other trials, and that change in EPA abundance in the placebo group was not correlated to clinical improvement [55]. Another trial [56] used a higher dose of ethyl-EPA (3 g/day for 12 weeks) which was compared to a liquid paraffin placebo administered to 40 medicated patients with schizophrenia. These authors reported a significant positive difference in symptom scores in the placebo and EPA groups after 3, 9 and 12 weeks. Moreover, in contrast to Peet and Horrobin [52], these researchers report that the largest reduction in PANSS scores is observed in those patients taking typical antipsychotics, not the atypical drug clozapine, although the difference did not reach statistical significance. It should be noted, however, that despite the fact that the authors' main *a priori *outcome measure was intended to be scores at 12 weeks, the difference between the two groups had in fact diminished by this time point. Unfortunately, the statistical analysis and trials outcomes were only reported in detail for the 9 week scores, although the authors do not highlight this point in their analysis. The failure to use the *a priori *outcome measure, along with deficits in other reporting aspects of the trial, diminishes our confidence in the robustness of this trial's findings.

In general, clinical trials of omega-3 fatty acids in schizophrenia, despite being mostly of high quality, have produced inconsistent results and small effects sizes of doubtful clinical significance. Although the trial by Peet and Horrobin [52] does report a significant improvement, this only occurs at one dose in a group of patients having a specific background treatment. Taken as a whole, the evidence suggests that omega-3 fatty acids little or no effect upon the symptoms of schizophrenia, and that even if a true effect exists, that it is unlikely to be clinically relevant.

### Personality disorders

Personality disorders (also known as axis-2 disorders) are a type of mental illness in which patients form inflexible patterns of actions and thoughts [12]. They are generally treated using various forms of psychotherapy although pharmacological treatments are commonly used for attending symptoms such as depression or anxiety. To date the efficacy of omega-3 PUFA has only been tested in one type of personality disorder, borderline personality disorder (BPD). Zanarini and Frankenburg [57] administered 1 g/day ethyl EPA or placebo to 30 subjects with the disorder for 8 weeks and assessed aggression and depression using the Modified Overt Aggression Scale (MOAS [58]) and the Montgomery-Asberg Depression Rating Scale (MADRS [59]) respectively. The authors' conclusion that "ethyl EPA was superior to placebo in diminishing aggression as well as the severity of depressive symptoms" is surprising given the data presented in the research report. For the MOAS the difference between change in baseline values between placebo and omega-3 PUFA groups was only -0.8 and for MADRS -1.5 which does not support the authors conclusions. Based on the published data we find that there is little evidence to support the use of omega-3 fatty acids in the treatment of BPD.

### Major depressive disorder

Major depressive disorder (MDD) is a serious affective illness [12] with a lifetime prevalence estimated to be between 5 and 11% of the population [60]. It is characterized by depressed mood, a loss of interest in pleasurable activities, changes in sleep patterns and weight, and reduced cognitive abilities and concentration. A significant proportion of persons (approximately 15%) with severe depression eventually commit suicide making it the seventh most frequent cause of death in the US [61]. Episodes can last up to several years and the illness has a high recurrence rate ranging from 50% after one episode to 90% for those with 3 or more occurrences [61]. Depression is most commonly treated with a variety of antidepressant drugs such as the selective serotonin reuptake inhibitors (SSRI) and tricyclic antidepressants. Most treatments have a response rate, defined as a 50% improvement in symptoms, of approximately 66%, and around 80–90% of patients eventually respond to some treatment [60]. However, a significant proportion (10 – 20%) of patients are treatment refractive i.e. they do not respond at all or respond poorly to therapy. Moreover, the proportion of successfully treated patients who stay well for even 18 months is disappointingly low, being around 70% [60]. These statistics indicate that there is much room for improvement in the treatment and prevention of depression. Such advances are difficult because the disease has a complex biological, psychological and sociological aetiology. Diet has been postulated to be involved and it has been argued that the rising rates of MDD [62] are at least partly due to a decreased consumption of omega-3 fatty acids, these having been replaced in the diet by omega-6 fatty acids [63,64]. In support of this hypothesis, studies of patients with major depressive disorder have shown a striking and consistent deficit in omega-3 fatty acid abundance, while omega-6 PUFA levels are largely unaffected [65]. These findings do not appear to be easily explained by such potentially confounding factors as antidepressant medication or smoking [48]. Epidemiological studies have suggested that omega-3 PUFA deficits may be due to decreased dietary intake, although metabolic dysfunction has also been postulated [65].

Such studies have lead several investigators to conduct clinical trials of omega-3 fatty acids in patients with depressive disorders (Table 4). The trials were not of a uniform quality, however, as assessed by Jadad scores which ranged from 2 to 5. Peet and Horrobin [66] carried out the first double-blind placebo-controlled trial testing omega-3 fatty acids in MDD. They administered 1, 2 or 4 g/day ethyl-EPA or a liquid paraffin placebo for 12 weeks to 70 patients with MDD who had persistent symptoms of depression despite the use of conventional anti-depressant drugs. They report that 1 g/day, but not 2 or 4 g/day ethyl-EPA, was significantly more likely than placebo to improve symptoms rated using the HDRS, the MADRS and the Beck Depression Inventory [67], with the effect being apparent at the earliest time point recorded, 4 weeks. The magnitude of the difference between omega-3 and placebo groups was, however, small. For example, ethyl EPA reduced the HDRS score by 3.8 points more than placebo. Peet and Horrobin [66] also report that a higher proportion of patients receiving 1 g/day achieved a 50% improvement in their symptoms (considered to be clinically significant) compared to the placebo group (9/17 vs. 5/17). The statistical significance of this difference was not reported, but can be calculated as being not significant (p = 0.163 for a two-sided test of proportions; p = 0.082 for the one-sided test).

**Table 4:** Double blind placebo-controlled trials of omega-3 fatty acids upon in mood disorders.

*Ref*	*Jadad score*	*Disorder*	*Meds*	*Age*	*Gen*	*Omega-3 fatty acid*	*Prep type*	*Duration*	*N*	*Outcome measure*	*Average baseline score*	Δ	*Statistical significant difference*
66	5	MDD	100%	50	81%	1 g/day EPA	Ethyl ester	12 weeks	34	HDRS	20	-3.8	Yes
						2 g/day EPA	Ethyl ester	12 weeks	35	HDRS	20	+0.3	No
						4 g/day EPA	Ethyl ester	12 weeks	34	HDRS	19	-0.3	No
68	3	MDD	100%	53	85%	2 g/day EPA	Ethyl ester	4 weeks	20	HDRS	22	-10.8	Yes
69	4	MDD	100%	38	80%	2.2 g/day EPA 1.1 g/day DHA	Crude mixture	8 weeks	22	HDRS	22	-7.2	Yes
71	4	CD	0%	10	25%	0.4 g/day EPA^1^ 0.2 g/day DHA	Crude mixture	16 weeks	20	CDRS	69	-25	Yes
74	2	MDD	0%	47	80%	2 g/day DHA	Crude mixture	6 weeks	35	HDRS	26	-2.3	No
75	3	MDD	79%	39	79%	0.6 g/day EPA 2.4 g/day DHA	Crude mixture	12 weeks	77	HDRS	12	+0.6	No
77	3	BD	100%	43	66%	6.2 g/day EPA 3.4 g/day DHA	Crude mixture	4 months	30	YMRS	7	+1.3	No
										HDRS	11	-7.7	Yes
80	5	BD	100%	47	76%	1 g/day EPA	Ethyl ester	12 weeks	50	YMRS	6	-3.6^2^	No
										HDRS	15	-3.6	No
						2 g/day EPA	Ethyl ester	12 weeks	51	YMRS	5	-1^2^	No
										HDRS	15	-3	No
						1 and 2 g/day groups combined	Ethyl ester	12 weeks	51	YMRS	6	-2.3^2^	No
										HDRS	15	-3.3	Yes
81	5	BD	100%	45	85%	6 g/day EPA	Ethyl ester	16 weeks	116	IDS	NS	-0.4	No
										YMRS	NS	+0.6	No
86	5	PPD	0%	31	100%	0.2 g/day DHA	Crude mixture	4 months	89	BDI	7	+0.4	No

MDD, CD, PPD and BD are major depressive disorder, childhood depression, post partum depression and bipolar disorder respectively. Δ refers to the difference between the change from baseline values for placebo and treatment groups (change from baseline symptom scores for the omega-3 fatty acid group minus change from baseline scores for the placebo group). Since for all scales zero is better, a negative value indicates a better than placebo response. Trial quality was assessed using Jadad design quality scores which can range from 0 to 5 with 5 being best. Outcome measures are HDRS: Hamilton Depression Rating Scale, YMRS: Young Mania Rating Scale, CDRS – Children's Depression Rating Scale, IDS – Inventory of Depressive Symptomatology. The average baseline score is the average of the omega-3 and placebo group scores at the start of the trial. Age refers to the mean age in years of the active and placebo groups. Gen (gender) refers to the % of female participants in the trial. Notes: ^1 ^Some children received a different capsule size resulting in a dose of 0.38 g EPA/day and 0.18 g DHA/day, ^2 ^Although the omega-3 fatty acid group performed better than placebo on the YMRS it should be noted that the mania symptoms worsened in both omega-3 PUFA and placebo groups during the trial.

An apparent antidepressant effect of ethyl-EPA was also reported by Nemets and colleagues [68]. These investigators used a 2 g/day dose of ethyl-EPA or undefined placebo for 4 weeks, administered to 20 medicated patients. They observed a significant reduction in symptoms measured using the HDRS in the EPA compared to the placebo group after 2 and 3 weeks of treatment as well as the main outcome measure duration of 4 weeks. The authors base their statistics on a per-protocol analysis which excludes participants who did not complete the trial, rather than the more acceptable intent-to-treat analysis. We have therefore performed such an analysis using the data contained in the report (Table 4) and find that the intent-to-treat analysis shows a slightly larger effect than the per-protocol analysis, which lends further support to the authors' conclusions. Furthermore, a significantly higher proportion of patients achieved a 50% reduction in symptoms in the EPA compared to the placebo groups (60% vs. 10% respectively). A beneficial effect of omega-3 fatty acids is also reported by Su and colleagues [69] who conducted a shorter duration 8 week double-blind placebo-controlled trial of 22 patients with MDD (all but 2 were currently medicated) using fish oil (which resulted in a daily dose of 2.2 g/day EPA and 1.1 g/day DHA) or an olive oil placebo. Of the patients completing the trial (the authors only provide the data for a per-protocol analysis; 4 placebo- and 2 intervention-allocated participants dropped out of the study), the omega-3 fatty acid group achieved a significantly greater reduction in depressive symptoms than the placebo group.

Most recently, omega-3 PUFA supplementation is reported to be beneficial in depression occurring in children. Childhood depression is less common than that in adults but is nevertheless estimated to affect 2 – 4% of children [70]. Nemets and colleagues [71] administered 400 mg EPA and 200 mg DHA daily (or 380 mg DHA and 180 mg DHA daily in a smaller capsule size) or a safflower oil placebo to 28 children who were currently depressed. The children were assessed every 4 weeks for 16 weeks using the Children's Depression Rating Scale (CDRS) [72] and Clinical Global Impression scale (CGI) [73]. The omega-3 PUFA group showed significant improvement compared to the placebo group using both the CDRS and GGI. The trial is also of note since the participants were not in receipt of conventional anti-depressant treatments although 5 were being treated for symptoms of ADHD with methylphenidate. One other trial has been conducted which also utilised an omega-3 PUFA as a monotherapy for depression (as opposed to being an adjunct to conventional antidepressants), although in this trial no beneficial effect of omega-3 PUFA supplementation was observed. Specifically, Marangell and colleagues [74] reported that 2 g/day DHA administered to 18 patients with MDD for 6 weeks did not improve symptom scores relative to 17 patients who received an undefined placebo. The trial differed from the previous studies reporting positive findings in that the authors used a preparation high in DHA, rather than high EPA content oils or ethyl-EPA. The nature of the DHA or placebo preparations are not described (contributing to a low Jadad score), although it is likely to that the former was a crude oil preparation given that 14 patients receiving DHA, but no patients in the placebo group, reported a "fish" aftertaste. A second trial reporting negative findings also utilised a DHA-rich oil [75] in which 77 patients with depression received either 2.4 g/day DHA and 0.6 g/day EPA or an olive oil placebo. Unlike the other trials described the authors included participants in the trial based on their HDRS score rather than by psychiatric interview and, as a consequence, the participants are likely to be more heterogenous in nature and may not have had true MDD according to DSM-IV criteria. Although a significant reduction in symptoms rated using the HDRS occurred in the omega-3 fatty acid group, no difference was apparent between placebo and omega-3 PUFA groups. It is important to note, however, that on average, the participants in this trial were only mildly depressed (baseline HDRS was 12 compared to an average of 21 for the other trials), which led to both the placebo and DHA groups achieving a cure rate (no significant depressive symptoms) approaching 100%. Such a high cure rate in both groups, whether because of a strong placebo effect or because of a lack of pathology at baseline, prevents us from drawing any firm conclusions regarding the efficacy of omega-3 PUFA in depression from this trial.

### Bipolar disorder

Like MDD, bipolar disorder (BD) is a disease of mood [12]. It is less prevalent than MDD, with a lifetime risk of 1–2%, and differs from MDD in that BD patients experience both depressive symptoms and emotional highs termed manic episodes [76]. Manic symptoms include inflated self-esteem that may reach delusional proportions, a decreased need for sleep, rapid speech and thoughts, and an inability to remain focused. BD is a serious and sometimes life threatening illness that is commonly treated using either lithium or anti-convulsant mood stabilizers such as valproic acid [6]. The investigation of omega-3 fatty acid abundance in BD is limited to only three studies which, although suggestive of a mild deficit of omega-3 PUFA in BD, are rather inconsistent and certainly not on par with the pronounced deficit observed in MDD [48]. Nevertheless, Stoll and colleagues [77] reported that, compared to those receiving an olive oil placebo, patients with BD who had received fish oils containing 6.2 g/day EPA and 3.4 g/day DHA for at least 30 days were significantly less likely to relapse (i.e. require changes in their treatment necessitating exit from the trial) (Table 4). The investigators also observed that the omega-3 fatty acid group had a better response than placebo in terms of depressive symptoms (rated using the HDRS) and overall pathology (using the Clinical Global Impression and Global Assessment Scales [78]), but not in terms of manic symptoms assessed using the Young Mania Rating Scale (YMRS [79]). Although small is size, the study did indicate a potentially beneficial effect of omega-3 fatty acids in BD, particularly in the depressive phase of the disorder, suggesting a similar mechanism to that which underlies the possible antidepressant effects in MDD. A depressive phase effect is also indicated by a later trial conducted by Frangou and colleagues [80]. These investigators conducted a high quality (Jadad score = 5) double-blind placebo-controlled trial of 75 medicated outpatients with bipolar disorder who were administered either 1 g or 2 g/day ethyl EPA or placebo for 12 weeks. They report that neither dose of EPA had any statistically significant effect upon mania (assessed using the YMRS), but both EPA doses resulted in a non-statistically significant decline in symptoms of depression (assessed using the HDRS). The authors report that by combining the two EPA groups and comparing them to the placebo group, then EPA shows a statistically significant effect upon depressive symptoms, but mania symptoms were still found to be unchanged. The need to combine the doses of EPA to obtain a statistically significant improvement is indicative of EPA having a rather small effect upon depressive symptoms. Additionally, it is worth noting that in MDD, the results of Peet and Horrobin dose-ranging trial indicated that an EPA dose of 2 g/day was *not *efficacious [66] (Table 4).

The most recent trial involving subjects with bipolar disorder focussed on two subgroups, those with a current major depressive episode and those with rapid-cycling illness (patients who switch quickly between manic and depressed states). Keck and colleagues [81] administered either 6 g/day ethyl-EPA or a liquid paraffin placebo to 95 patients with bipolar depression or 86 patients with rapid cycling bipolar disorder. The authors report no benefit of omega-3 PUFA treatment relative to the placebo for either mania or depression, assessed using the YMRS and the Inventory of Depressive Symptomatology respectively [82], or on time to withdrawal from the trial. Data are presented only for the results of the two bipolar groups combined although the report states that the diagnostic subtype i.e. depressed and rapid cycling did not affect treatment response. Whiles this precludes any further analysis of the treatment effects upon bipolar subtypes, the data reported are comparable to the other bipolar disorder trials since they also did not differentiate by subtype. The dose of EPA used in the trial by Keck and colleagues (6 g/day) was approximately the same as that used in the Stoll and colleagues trial, but differed in that Keck and colleagues administered purified ethyl-EPA [81] while Stoll and colleagues co-administered EPA with 3.4 g/day DHA. Given that higher doses of purified EPA were found to be ineffective in MDD [66], it is possible that the negative data reported by Keck and colleagues [81] is due to using too high a daily dose of the lipid in the absence of sufficient quantities of DHA or other component of the crude oil. Finally, a fourth placebo controlled trial of omega-3 PUFA in bipolar disorder has been published [83]. This trial was, however, limited to reports of compliance and adverse events with no psychopathology being included. We therefore did not consider this trial further.

The results of the bipolar disorder trials are somewhat supportive of an anti-depressant effect of omega-3 fatty acids similar to that observed in MDD, although one trial did reported omega-3 PUFA to be ineffective. One concern of treating depression in patients with bipolar disorder is that the therapy will exacerbate manic episodes. However none of the trials indicated that omega-3 PUFA has any effect upon mania, either negative or positive. Since all trials utilized supplements containing predominantly EPA, no conclusions can be drawn with respect to the relative efficacy in BD, if any, of the various species of omega-3 PUFA.

### Post-partum depression

There is evidence of omega-3 PUFA deficiency in women who develop postpartum depression (PPD) [84]. PPD is a serious mood disorder with the same symptoms as MDD but which occurs in the postpartum period. Women who developed PDD are reported to have decreased levels of omega-3 fatty acids, but normal omega-6 fatty acid abundance [84]. This finding is particularly interesting because the participants had reduced omega-3 fatty acid abundance prior to the onset of depression, indicating that omega-3 fatty acid deficiency may be predictive for the occurrence of depression, and suggesting the possible utility of early nutritional and/or conventional pharmacological intervention to reduce the risk of PPD. The use of omega-3 fatty acids as an antidepressant in pregnancy would be attractive given the growing evidence that conventional antidepressants may have an adverse effect upon the child [85]. This, along with the promising results of omega-3 fatty acid therapy obtained in MDD, has prompted researchers to investigate their use in PPD. One group of investigators [86] administered 200 mg/day of DHA in the form of algal triglycerides or an undefined placebo to 138 pregnant women using a randomized double blind design. Depressive symptoms were monitored using the self-rated Beck Depression Inventory before and 3 weeks, 2 months and 3 months after delivery. The Beck scale is not a diagnostic instrument and only a subset of participants were assessed for present and past incidence of MDD using a structured clinical interview. The study reported that DHA treatment did not differ from placebo in terms of depressive symptoms assessed using the Beck scale, leading the authors to conclude that DHA does not reduce the incidence of PPD. Furthermore, among the patients who underwent the structured clinical interview, the incidence of current depression did not differ between the DHA and placebo groups [86]. It should be noted, however, that the women studied had, as a group, minimal depressive symptoms at the start of the trial and only a small proportion actually became depressed. As such the trial does not answer the questions of whether administration of omega-3 PUFA may be useful once a woman has actually developed PPD. Furthermore, given the results obtained with MDD, it is possible that 200 mg/day DHA is insufficient for even prophylactic use. Treatment of diagnosed PPD with omega-3 fatty acids has been studied using two open-label studies that reported both positive [87] and negative results [88]. A placebo-controlled trial utilizing higher doses of omega-3 fatty acids would be of great interest.

### Meta-analysis of effect upon depression

**Figure 2:**
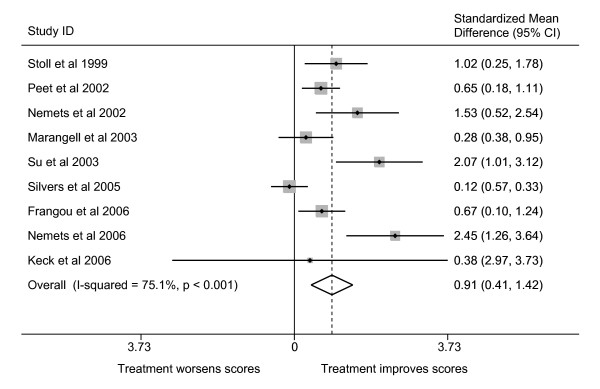
Standardized mean differences in depressive symptoms in omega-3 PUFA-treated compared to placebo-treated subjects with major depressive disorder or bipolar disorder. SMD are the standardized mean differences (scaled to standard deviations) in the change-from-baseline scores in the placebo and treatment groups, along with 95% confidence intervals. A summary of each study's characteristics can be found in Table 4. All studies rated patients using the HDRS, except that conducted by Llorente and colleagues [73], which was therefore excluded from the analysis. I-squared is the proportion of variance attributable to the heterogeneity between studies, and the p-value is for Cohen's Q, a test of the statistical significance of that heterogeneity. The overall estimate is the inverse-variance-weighted average of the studies based on a random effects model.

The use of the comparable ratings of depression by most investigators allowed us to compare the trials to determine if there was evidence that omega-3 PUFA had a significant mood-elevating effect in either MDD or bipolar disorder. Combining all of the MDD and bipolar trials using meta-analytical techniques shows that omega-3 fatty acid supplementation is significantly more effective than placebo in the treatment of depression (Figure 2). The magnitude of that effectiveness is approximately 0.91 standard deviations of improvement, given the characteristics of the populations studied to date and noted above. Such a finding is in line with a previously reported meta-analysis which analysed a smaller number of trials [89]. There was, however, significant heterogeneity between the studies. We noted three possible factors that might influence that heterogeneity (baseline HDRS score, depression type and predominant omega-3 PUFA species) and tested their effects on the overall estimate using random effects meta-regression (Table 5). This analysis showed that the primary factor responsible for heterogeneity in the combined effect estimate is the PUFA species utilised. When PUFA species is taken into account the amount of between-study variance (τ^2^) is markedly reduced. The predicted overall standardised mean difference (i.e. SMD: the mean difference between PUFA and placebo groups scaled to the standard deviation) of EPA or a predominantly EPA mixture were used would be 1.18, whereas for a DHA or predominantly DHA mixture, the predicted overall SMD would be 0.06 (Wald test for difference: p = 0.007). This suggests that EPA, rather than DHA, is efficacious in the alleviating the symptoms of depression, with an SMD value suggesting that EPA has a major effect upon the symptoms of depression. Two points should be note however. Firstly, as we have previously discussed, the negative DHA trial conducted by Silvers and colleagues [75] may have encountered a ceiling effect in terms of antidepressant action. Secondly, Marangell and colleagues [74] utilised DHA as monotherapy whereas adjunct omega-3 PUFA therapy was used in the other adult depression trials. Thus, an alternative explanation for the lack of observed efficacy is that omega-3 fatty acids (DHA or EPA) may only be effective when given in combination with other antidepressant medications, possibly by altering the pharmacokinetics of the other drugs. On the other hand, EPA administered in the absence of conventional antidepressants did show an antidepressant effect in childhood depression suggesting that EPA does indeed have an intrinsic efficacy. Thus although the available evidence is suggestive that EPA plays a more significant role in the antidepressant effect of omega-3 PUFA than does DHA, until a trial specifically designed to test this hypothesis is conducted this will remain an open question. Moreover, the lack of an antidepressant effect of EPA administered in purified form in bipolar depression should temper any move to conclude that EPA is solely responsible for any possible efficacy.

**Table 5:** Meta-regression to identify factors that account for significant heterogeneity in the overall standardized mean difference (SMD) between PUFA treatment and placebo upon depression in patients with major depressive disorder or bipolar disorder.

**Factor in Model**	**Factor category**	**Between-study variance (τ**^2^)	**SMD for category**	**P(difference = 0)**
No factor		0.39	0.91	NA
Baseline mean HDRS score	<17	0.31	0.48	0.291
	>17		1.01	
Depression type	Bipolar	0.48	0.80	0.761
	MDD		0.98	
PUFA species utilised	Predominantly DHA	0.18	0.06	0.009
	Predominantly EPA		1.18	

Meta-regression models the effect of study variables on the between-study variance (τ^2^) in a random effects meta-analysis. To conserve degrees of freedom, each factor was tested independently. A smaller τ^2 ^compared to the no-factor base model indicates that the factor explains more between-study variance. A meta-regression model also allows us to calculate a Wald test for the difference in the SMD for each category of the explanatory factor. A p-value less than 0.05 indicates that there is a statistically significant difference between the SMD for each category. All trials listed in Table 4 were included in the analysis of PUFA species utilised and depression type. For those studies using the HDRS, we used a baseline mean depression score of 17 as the cut-off value for moderate vs. mild depression [89]. The analysis of baseline depression scores excluded two trials – the trial conducted by Keck and colleagues [81] who did not report baseline scores, and the trial by Nemets and colleagues [71] because it used the Children's Depression Rating Scale and there is no agreement on what score constitutes moderate depression. NA – not applicable.

### Possible mechanisms underlying the psychoactive effects of omega-3 PUFA

The clinical trials data is therefore supportive of n-3 PUFA playing a significant role in mood disorders, while providing weaker support for their ability to enhance attention and reduce anxiety, and little or no support for the amelioration of the symptoms of schizophrenia. Furthermore, for mood disorders, there is some evidence that EPA is more efficacious then DHA in reducing the symptoms of depression. We will now briefly consider whether the psychoactive characteristics of omega-3 PUFA are biologically plausible from a neuroscientific perspective, and how the findings emanating from the clinical trials might be explained [6]. A useful means to consider these questions is to utilize the insight gained from almost six decades of use and study of existing pharmacological interventions. For mood and/or anxiety disorders current treatments include compounds which enhance either serotonergic neurotransmission e.g. SSRI, noradrenergic neurotransmission e.g. selective noradrenergic reuptake inhibitors, monoamine oxidase inhibitors, or both e.g. tricyclic antidepressants [6]. These drugs are thought to exert their beneficial effects by increasing the influence that neurons, originating in the locus coeruleus or dorsal raphe, have upon their targets in the limbic system, thalamus and prefrontal cortex [6]. The efficacy of these drugs is thought to derive from their ability to enhance serotonergic or dopaminergic neurotransmission, whereas antagonism of specific receptors of serotonin, dopamine or acetylcholine by some compounds are believed to be responsible for their unwanted side-effects [6]. In the case of attentional disorders the treatment of choice is methyphenidate. This compound most likely acts as a form of cognitive enhancer by means of increasing dopaminergic neurotransmission within the mesocortical dopamine system which projects from the ventral tegmentum in the midbrain to the prefrontal cortex [6]. In the case of schizophrenia, however, medications are thought to work by doing the opposite, that is, by reducing rather than enhancing dopaminergic neurotransmission. Thus, both typical and atypical antipsychotic drugs are thought to diminish the positive symptoms of schizophrenia by antagonizing dopamine D2 receptors within the mesolimbic dopamine system, a projection which originates in the mid brain and terminates in the nucleus accumbens and amygdala [6]. Moreover, agents which increase dopaminergic neurotransmission can induce psychosis in healthy individuals and actually worsen symptoms in patients with schizophrenia [6]. In addition, some evidence suggests that the same dopaminergic agents that worsen symptoms of schizophrenia possess antidepressant properties although this remains a matter of some controversy [90]. It would therefore be very surprising indeed if any medicinal compound was found to be a beneficial treatment for the core symptoms of both schizophrenia *and *mood disorders. Moreover, if omega-3 PUFA were found to enhance dopaminergic and/or serotonergic neurotransmission, then the existing pharmacological data would predict that they should be effective in the treatment of mood, attentional and anxiety disorders. The literature is, however, ambivalent. An extensive series of experiments conducted by Chalon and colleagues [91] has indicated that omega-3 PUFA deficiency beginning in utero results in a reduced ability of neurons to (i) release dopamine in the frontal cortex and nucleus accumbens in response to tyramine, and (ii) release serotonin in the hippocampus in response to fenfluramine [92,93]. Moreover, n-3 PUFA deficiency in rats can result in altered cortical dopamine D2 receptor density, a decreased number of dopamine containing vesicles in each synapse, decreased binding to the vesicular monoamine transporter and increased dopamine metabolism, all of which are indicative of decreased dopaminergic activity [93-95]. Such findings do suggest that supplementation with omega-3 PUFA should enhance, or restore, dopaminergic and/or serotonergic neurotransmission, and are hence supportive of a significant role for omega-3 PUFA in mood, anxiety and attentional disorders, but, importantly, not in schizophrenia. It should be noted, however, that the model used by Chalon and colleagues [91] results in a much more severe omega-3 PUFA deficiency, starting in utero, than is observed in, for example, patients with mood disorders [48,65]. Moreover, animal behavioural data is not consistent with the anti-dopaminergic effect of omega-3 PUFA deficiency suggested by the neurochemical investigations [96]. Using a similar model to Chalon and colleagues [91], but which resulted in a milder omega-3 PUFA deficiency, Levant and colleagues reported that rats deficient in omega-3 PUFA in utero and after birth exhibited increased basal locomotor activity and decreased catalepsy in response to haloperidol [96], both of which are suggestive of elevated, rather than decreased, basal dopaminergic activity in omega-3 PUFA deficient animals. Indeed, these authors observed that returning animals to a normal, non-omega-3 PUFA deficient diet, reversed some of the abnormalities they detected i.e. they increased haloperidol-induced catalepsy. The discrepancy between the two animal models may be due to hypersensitivity of dopamine D2 receptors in the mesolimbic dopamine system caused by decreased dopamine availability occurring subsequent to the omega-3 PUFA deficiency [96]. Dopamine receptor sensitivity may then overcome the effects of decreased dopamine abundance resulting in increased locomotor activity. While this is a reasonable explanation, the discrepancy between behavioural and neurochemical results makes it impossible to extend these animal model findings, with any certainty, to human psychiatric illness. On the other hand, the results reported by Levan and colleagues [96] would predict that omega-3 PUFA should diminish symptoms of schizophrenia by means of reducing dopaminergic neurotransmission, and exacerbate attentional deficits by the same mechanism. Since it is clear from the clinical trial data that this does not happen, we are forced to conclude that the model Levan and colleagues [96] used is not useful as a means to understand the potential psychoactive effects of omega-3 PUFA supplementation (although that does not diminish the insight given into how omega-3 PUFA deficiency in utero may play a role in the aetiology of mental illness). It is, however, worth repeating, that an enhancement of serotonergic, and possibly dopaminergic neurotransmission, by omega-3 PUFA is most consistent with the results of the existing clinical trials of omega-3 PUFA in mood disorders. Further progress in elucidating the mechanism of omega-3 PUFA action is likely to come from investigations using positron emission tomography imaging which, with the selection of appropriate ligands, can be used to assess dopaminergic and serotonergic function in the brain of patients [97].

At the biochemical level how might omega-3 PUFA deficiency and supplementation affect the nervous system? The conventional view [2] of omega-3 PUFA bioactivity is that they act as negative regulators of the eicosanoid signaling systems dependent upon the metabolism of the omega-6 PUFA, AA e.g. the prostaglandin, leukotriene and HETE pathways. Specifically, dietary supplementation with omega-3 PUFA is thought to increase the abundance of omega-3 PUFA in membrane phospholipids which can then inhibit AA-dependent signalling either directly, by replacing AA as the eicosanoid substrate of cyclooxygenases and lipoxygenases, or indirectly by altering the expression of proteins involved in the signalling cascade. Given that the metabolites of AA have diverse effects within the brain, including playing a role in dopaminergic and serotonergic signaling systems, such an action by omega-3 PUFA has been suggested to have important consequences for brain physiology and behaviour [7]. For example, agonists of dopamine D2 and D4 receptors activate AA-dependent signaling [98,99], while the production of free AA and AA metabolites in response to serotonin 5HT2 receptor occupancy is thought to play a key role in the mechanism of SSRI action [100,101]. Although limited, such evidence suggests the use of AA-dependent signaling by the neurotransmitter systems known to be stimulated by existing treatments for mood, anxiety and attentional disorders. Such data is not supportive of a mechanism which postulates that omega-3 PUFA actually *inhibits *this signaling pathway. Furthermore, although EPA is a minor brain fatty acid compared to the abundant DHA [102], both omega-3 PUFA are equally effective inhibitors of AA-derived eicosanoid synthesis [103], a finding which is at odds with the observation that EPA may be more clinically efficacious in the treatment of mood disorders.

Differential effects of EPA and DHA have, however, been reported (for example see [104] and [105]). Of interest is an *in vitro *observation obtained using the U937 monocyte derived cell line which indicates that long term supplementation with EPA can increase the stimulated release of AA from membrane phospholipids, whereas DHA has no effect [106]. This role of EPA was likely due to it inhibiting the re-uptake of AA to reform an intact phospholipid, a two enzyme metabolic pathway which acts to reverse the hydrolysis of the AA-containing phospholipid by phospholipase A_2 _[107]. Although the biochemical mechanism is not fully understood, it is likely that EPA can inhibit the expression and/or activity of the enzymes lysophospholipid acyltransferase (LPAT) or fatty acid CoA ligase (FACL), both of which are required for reuptake of AA to occur [107]. Indeed, reduced activity of FACL appears to enhance AA-dependent signaling reactions since reduced reuptake will increase the supply of AA available for eicosanoid synthesis [108]. Such a mechanism is of interest given that it provides a means by which AA-dependent signaling could be enhanced by its 20 carbon omega-3 PUFA counterpart, EPA, a characteristic which, as we have argued, may enhance dopaminergic and serotonergic neurotransmission.

Stimulation of AA-dependent signaling by EPA has also been suggested by Horrobin and colleagues, but via a different mechanism: the ability of omega-3 PUFA to increase the abundance of AA in membrane phospholipids [109]. This again goes against conventional thinking which maintains that omega-3 PUFA are antagonists of AA-dependent processes. In what may prove to be an important observation, these authors observed that feeding rats a diet containing 0.01% or 0.1% EPA increased levels of DHA, EPA *and *AA, whereas a 1% EPA diet increased DHA and EPA but *decreased *AA levels in erythrocytes [109]. A similar relationship between EPA dose and AA levels was also noted in patients with schizophrenia who after receiving 1 g/day and 2 g/day EPA had elevated erythrocyte AA levels, whereas 4 g/day EPA decreased erythrocyte AA levels [109]. It is likely that a reduction in AA abundance at higher EPA doses is due to significant inhibition of AA uptake into the membrane, possibly occurring via the inhibition of LPAT as described earlier [106]. The mechanism by which low dose EPA increases AA levels is unknown, but there is evidence that EPA can elevate the activity of one of the other enzyme involved in AA uptake, FACL [110]. As such, EPA and potentially other omega-3 PUFA, appear to have two distinct cellular effects (and biochemical targets) with respect to phospholipid metabolism: at low doses they may enhance incorporation of both themselves *and *omega-6 PUFA into the membrane, but at higher doses they *decrease *omega-6 incorporation. It is of interest to note that of the two clinical trials examining the ability of omega-3 PUFA to ameliorate attentional deficits which reported a significant improvement in symptoms, in both cases a combination of omega-3 and omega-6 PUFA were administered [29,32]. It would also be of interest to investigate the possibility that omega-3 and omega-6 PUFA may act in a synergistic (rather than antagonistic) manner in future clinical trials. In addition, if enhancement of AA-dependent signaling is the underlying mechanism of low dose EPA action in mood disorders then it would be expected that higher doses of EPA, which are associated with decreased AA abundance [109], would have a lesser antidepressant effect. Some evidence supports this hypothesis given that 4 g/day or higher EPA is reported to lack mood elevating activity in both major depressive disorder and bipolar disorder [66,81]. On the other hand, high dose EPA was found to reduce the symptoms of depression in another trial involving patients with bipolar disorder [77], although in this trial EPA was co-administered with DHA. To understand why adding DHA to EPA may be more effective than EPA alone, especially at high doses, it is worth remembering that EPA abundance in the brain (approximately 0.2% of fatty acids) is much lower than DHA (approximately 15% of fatty acids) [111,112]. Although supplementation with EPA does not change the fact that DHA predominates in the brain, such supplementation does have a much larger relative effect upon EPA abundance compared to DHA abundance [111]. Thus, as EPA intake increases, the ratio of DHA to EPA in the brain markedly falls. Since it is only recently that purified EPA has been available for human consumption, little is known regarding the consequences for brain function that administering high doses of EPA in the absence of DHA may have. Although it is tempting to attempt to extend the high purity, single compound norm of the pharmaceutical industry to the field of nutrition, the clinical trials data should alert us to the possibility that administration of purified EPA alone may not in fact be the most clinically effective formulation, and that the addition of DHA, AA or other fatty acids could actually be more beneficial for the patient. Such a hypothesis is testable using randomised clinical trials which compare the relative efficacy of placebo, EPA, EPA/DHA and other PUFA combinations.

Besides their role as modulators of AA-dependent signaling, it is emerging that both EPA and DHA are precursors of bioactive metabolites in their own right. For example, EPA can be metabolised by cyclooxygenase to form the 3-series prostaglandins [113]. Most recently, a new class of compounds have been identified termed resolvins, with EPA and DHA giving rise to the E- and D-series resolvins respectively [114]. The resolvins appear to act via cell surface receptors and are thought to have an important anti-inflammatory role [114]. It has been confirmed that they are formed in the nervous system but besides having a role in reducing neuroinflammation their function remains unknown [115,116]. Given that differences in function between the EPA- and DHA-derived compounds have already been identified [114], the possibility must be considered that one or more of these compounds may mediate the psychoactive effects of EPA, and that drugs designed to target their receptors may be therapeutically useful as treatments for mental illness.

## Conclusion

The available data are not supportive of omega-3 PUFA being efficacious in the treatment of schizophrenia or borderline personality disorders, while the investigation of their use in anxiety disorders is at an early stage. Of all the illnesses considered, the strongest evidence supports the use of omega-3 PUFA in the treatment of mood disorders with approximately 1 – 2 g/day of omega-3 PUFA appearing to be effective. An ability of omega-3 PUFA to treat attentional deficits is suggested by some trials although strong evidence is lacking. Larger trials will hopefully be conducted which allow more definitive statements to be made regarding the efficacy of omega-3 PUFA in these disorders over longer time periods, as well as determining the most beneficial omega-3 PUFA formulation to use.

All of the clinical trials described in this review had one conclusion in common – that omega-3 fatty acids had no serious side effects (loose stool were sometimes, though rarely, reported). These compounds, therefore, represent an attractive and novel treatment modality. Although the clinical trials varied in quality of design and tended to be small in terms of participants, further investigation of the use of omega-3 PUFA to treat at least some mental health problems is warranted. Many recent trials have utilised omega-3 formulations containing a high proportion of EPA compared to other omega-3 types such as DHA. We did find some evidence to support the use of such a high-EPA formulation for treatment of depressive symptoms although more study of the possibly differential efficacies of omega-3 PUFA species is required. Moreover, it should be noted that some evidence suggests that the use of formulations containing only EPA and lacking any DHA may actually be less effective than an EPA/DHA mixture, and that combinatorial therapies which include mixtures of both omega-3 and omega-6 PUFA should be considered [81]. This may relate to the fact that the brain contains predominantly DHA and the used of high levels of EPA may have unforeseen negative consequences especially when used over longer time periods.

Overall, based on the available evidence, it is not possible to firmly recommend omega-3 PUFA as either sole or adjunctive treatment for any of the mental illnesses considered. On the other hand, the research work done to date is certainly exciting and offers a strong foundation on which to base future clinical investigations.

## Competing interests

The author(s) declare that they have no competing interests.

## Authors' contributions

BR, JS and LS all contributed to the collation of trials data and their critical review. LS performed the meta-analysis. All authors have read and approved the final manuscript.

## References

[B1] Emken EA, Adlof RO, Gulley RM (1994). Dietary linoleic acid influences desaturation and acylation of deuterium-labeled linoleic and linolenic acids in young adult males. Biochim Biophys Acta.

[B2] Holub DJ, Holub BJ (2004). Omega-3 fatty acids from fish oils and cardiovascular disease. Mol Cell Biochem.

[B3] Peet M, Brind J, Ramchand CN, Shah S, Vankar GK (2001). Two double-blind placebo-controlled pilot studies of eicosapentaenoic acid in the treatment of schizophrenia. Schizophr Res.

[B4] Simopoulos AP (1991). Omega-3 fatty acids in health and disease and in growth and development. Am J Clin Nutr.

[B5] von Schacky C (2004). Omega-3 fatty acids and cardiovascular disease. Curr Opin Clin Nutr Metab Care.

[B6] Stahl SM (2000). Essential psychopharmacology Neuroscientific basis and practical applications.

[B7] Glen AIM, Ross BM, Curtis-Prior P (2004). Eicosanoids in the central nervous system. The Eicosanoids.

[B8] Carlezon surWA Jr, Mague SD, Parow AM, Stoll AL, Cohen BM, Renshaw PF (2005). Antidepressant-like effects of uridine and omega-3 fatty acids are potentiated by combined treatment in rats. Biol Psychiatry.

[B9] Egger M, Davey Smith G, Altman DG (2001). Systematic reviews in health care: meta-analysis in context.

[B10] Jadad AR, Moore RA, Carroll D, Jenkinson C, Reynolds DJM, Gavaghan DJ, McQuay HJ (1996). Assessing the quality of reports of randomized clinical trials: Is blinding necessary?. Control Clin Trials.

[B11] Szatmari P (1992). The validity of autistic spectrum disorders: a literature review. J Autism Dev Disord.

[B12] American Psychiatric Association (1994). Diagnostic and statistical manual of mental disorders.

[B13] Doggett AM (2004). ADHD and drug therapy: is it still a valid treatment?. J Child Health Care.

[B14] Richardson AJ, Puri BK (2000). The potential role of fatty acids in attention-deficit/hyperactivity disorder. Prostaglandins Leukot Essent Fatty Acids.

[B15] Richardson AJ, Ross MA (2000). Fatty acid metabolism in neurodevelopmental disorder: a new perspective on associations between attention-deficit/hyperactivity disorder, dyslexia, dyspraxia and the autistic spectrum. Prostaglandins Leukot Essent Fatty Acids.

[B16] Mitchell EA, Aman MG, Turbott SH, Manku M (1987). Clinical characteristics and serum essential fatty acid levels in hyperactive children. Clinical Pediatrics.

[B17] Stevens LJ, Zentall SS, Deck JL, Abate ML, Watkins BA, Lipp SR (1995). Essential fatty acid metabolism in boys with attention-deficit hyperactivity disorder. Am J Clin Nutr.

[B18] Bekaroglu M, Aslan Y, Gedik Y, Deger O, Mocan H, Erduran E, Karahan C (1996). Relationships between serum free fatty acids and zinc, and attention deficit hyperactivity disorder: a research note. J Child Psychol Psychiatry.

[B19] Burgess JR, Stevens L, Zhang W, Peck L (2000). Long-chain polyunsaturated fatty acids in children with attention-deficit hyperactivity disorder. Am J Clin Nutr.

[B20] Ross BM, McKenzie I, Glen I, Bennett CP (2003). Increased levels of ethane, a non-invasive marker of n-3 fatty acid oxidation, in breath of children with attention deficit hyperactivity disorder. Nutr Neurosci.

[B21] Aman MG, Mitchell EA, Turbott SH (1987). The effects of essential fatty acid supplementation by Efamol in hyperactive children. J Abnorm Child Psychol.

[B22] Arnold LE, Kleykamp D, Votolato NA, Taylor WA, Kontras SB, Tobin K (1989). Gamma-linolenic acid for attention-deficit hyperactivity disorder: placebo-controlled comparison to D-amphetamine. Biol Psychiatry.

[B23] Joshi K, Lad S, Kale M, Patwardhan B, Mahadik SP, Patni B, Chaudhary A, Bhave S, Pandit A (2006). Supplementation with flax oil and vitamin C improves the outcome of Attention Deficit Hyperactivity Disorder (ADHD). Prostaglandins Leukot Essent Fatty Acids.

[B24] Fontani G, Corradeschi F, Felici A, Alfatti F, Migliorini S, Lodi L (2005). Cognitive and physiological effects of Omega-3 polyunsaturated fatty acid supplementation in healthy subjects. Eur J Clin Invest.

[B25] Voigt RG, Llorente AM, Jensen CL, Fraley JK, Berretta MC, Heird WC (2001). A randomized, double-blind, placebo-controlled trial of docosahexaenoic acid supplementation in children with attention-deficit/hyperactivity disorder. J Pediatr.

[B26] Achenbach TM, Edelbrock C (1983). Manual for the child behavior checklist and revised child behavior profile.

[B27] Conners CK (1999). Clinical use of rating scales in diagnosis and treatment of attention-deficit/hyperactivity disorder. Pediatr Clin North Am.

[B28] Hirayama S, Hamazaki T, Terasawa K (2004). Effect of docosahexaenoic acid-containing food administration on symptoms of attention-deficit/hyperactivity disorder – a placebo-controlled double-blind study. Eur J Clin Nutr.

[B29] Richardson AJ, Puri BK (2002). A randomised double-blind, placebo-controlled study of the effects of supplementation with highly unsaturated fatty acids on ADHD-related symptoms in children with specific learning difficulties. Prog Neuropsychopharm Biol Psychiatry.

[B30] Stevens L, Zhang W, Peck L, Kuczek T, Grevstad N, Mahon A, Zentall SS, Arnold LE, Burgess JR (2003). EFA supplementation in children with inattention, hyperactivity, and other disruptive behaviors. Lipids.

[B31] Mendelsohn M, Erdwins C (1978). The Disruptive Behavior Scale: an objective assessment of unmanageable social behavior in adolescents. J Clin Psychol.

[B32] Richardson AJ, Montgomery P (2005). The Oxford-Durham study: a randomized, controlled trial of dietary supplementation with fatty acids in children with developmental coordination disorder. Pediatrics.

[B33] Dewey D, Wilson BN (2001). Developmental coordination disorder: what is it?. Phys Occup Ther Pediatr.

[B34] Amminger GP, Berger GE, Schafer MR, Klier C, Friedreich MH, Feucht M (2007). Omega-3 fatty acids supplementation in children with autism: a double-blind randomized, placebo-controlled pilot study. Biol Psychiatry.

[B35] Aman MG, Singh NN, Stewart AW, Field CJ (1985). The aberrant behaviour checklist: a behaviour chekclist for the assessment of treatment effects. Am J Ment Defic.

[B36] Bell J, MacKinlay EE, Dick JR, MacDonald DJ, Boyle RM, Glen AC (2004). Essential fatty acids and phospholipase A_2 _in autistic spectrum disorders. Prostaglandins Leukot Essent Fatty Acids.

[B37] Fux M, Benjamin J, Nemets B (2004). A placebo-controlled cross-over trial of adjunctive EPA in OCD. J Psychiatr Res.

[B38] Goodman WK, Price LH, Rasmussen SA, Mazure C, Fleischmann RL, Hill CL, Heninger GR, Charney DS (1989). The Yale-Brown Obsessive Compulsive Scale. I. Development, use, and reliability. Arch Gen Psychiatry.

[B39] Hamilton M (1960). A rating scale for depression. J Neurol Neurosurg Psychiatry.

[B40] Bruss GS, Gruenberg AM, Goldstein RD, Barber JP (1994). Hamilton Anxiety Rating Scale Interview guide: joint interview and test-retest methods for interrater reliability. Psychiatry Res.

[B41] Buydens-Branchey L, Branchey M (2006). n-3 polyunsaturated fatty acids decrease anxiety feelings in a population of substance abusers. J Clin Psychopharmacol.

[B42] McNair DM, Lorr M, Droppleman LF (1971). Manual for the Profile of Mood States.

[B43] Yehuda S, Rabinovitz S, Mostofsky DI (2005). Mixture of essential fatty acids lowers test anxiety. Nutr Neurosci.

[B44] Hamazaki K, Itomura M, Huan M, Nishizawa H, Sawazaki S, Masatoshi T, Watanabe S, Hamazaki T, Terasawa K, Yazawa K (2005). Effect of omega-3 fatty acid-containing phospholipids on blood catecholamine concentrations in healthy volunteers: a randomized, placebo-controlled, double-blind trial. Nutrition.

[B45] Green P, Hermesh H, Monselise A, Marom S, Presburger G, Weizman A (2006). Red cell membrane omega-3 fatty acids are decreased in nondepressed patients with social anxiety disorder. Eur Neuropsychopharmacol.

[B46] Mueser KT, McGurk SR (2004). Schizophrenia. Lancet.

[B47] Horrobin DF (1998). The membrane phospholipid hypothesis as a biochemical basis for the neurodevelopmental concept of schizophrenia. Schizophr Res.

[B48] Ross BM, Sieswerda LE, Teale MC (2006). Omega-3 fatty acids in affective and psychotic disorders. Omega-3 fatty acid research.

[B49] Joy CB, Mumby-Croft R, Joy LA (2006). Polyunsaturated fatty acid supplementation for schizophrenia. Cochrane Database Syst Rev.

[B50] Kay SR, Fiszbein A, Opler LA (1987). The positive and negative syndrome scale (PANSS) for schizophrenia. Schizophr Bull.

[B51] Hunter RH, Gilbody SM, Joy CB, Kennedy E, Song F (2003). Risperidone versus typical antipsychotic medication for schizophrenia. Cochrane Library.

[B52] Peet M, Horrobin DF (2002). A dose-ranging exploratory study of the effects of ethyl-eicosapentaenoate in patients with persistent schizophrenic symptoms. J Psychiatr Res.

[B53] Fenton WS, Dickerson F, Boronow J, Hibbeln JR, Knable M (2001). A placebo-controlled trial of omega-3 fatty acid (ethyl eicosapentaenoic acid) supplementation for residual symptoms and cognitive impairment in schizophrenia. Am J Psychiatry.

[B54] Horrobin DF (2003). Omega-3 fatty acid for schizophrenia. Am J Psychiatry.

[B55] Fenton WS, Dickerson F, Boronow J, Hibbeln JR, Knable MB (2003). Reply to: Omega-3 fatty acid for schizophrenia. Am J Psychiatry.

[B56] Emsley R, Myburgh C, Oosthuizen P, van Rensburg SJ (2002). Randomized, placebo-controlled study of ethyl-eicosapentaenoic acid as supplemental treatment in schizophrenia. Am J Psychiatry.

[B57] Zanarini MC, Frankenburg FR (2003). Omega-3 fatty acid treatment of women with borderline personality disorder: a double-blind, placebo-controlled pilot study. Am J Psychiatry.

[B58] Ratey JJ, Gutheil CM (1991). The measurement of aggressive behavior: reflections on the use of the Overt Aggression Scale and the Modified Overt Aggression Scale. J Neuropsychiatry Clin Neurosci.

[B59] Montgomery SA, Asberg M (1979). A new depression scale designed to be sensitive to change. Br J Psychiatry.

[B60] McIntyre RS, O'Donovan C (2004). The human cost of not achieving full remission in depression. Can J Psychiatry.

[B61] Chen YW, Dilsaver SC (1996). Lifetime rates of suicide attempts among subjects with bipolar and unipolar disorders relative to subjects with other Axis I disorders. Biol Psychiatry.

[B62] Klerman GL, Weisman MM (1989). Increasing rates of depression. JAMA.

[B63] Smith RS (1991). The macrophage theory of depression. Med Hypotheses.

[B64] Hibbeln JR (1998). Fish consumption and major depression. Lancet.

[B65] Ross BM (2007). Omega-3 fatty acid deficiency in major depressive disorder is caused by the interaction between diet and a genetically determined abnormality in phospholipid metabolism. Med Hypotheses.

[B66] Peet M, Horrobin DF (2002). A dose-ranging study of the effects of ethyl-eicosapentaenoate in patients with ongoing depression despite apparently adequate treatment with standard drugs. Arch Gen Psychiatry.

[B67] Beck AT, Ward CH, Mendelson M, Mock J, Erbaugh J (1961). An inventory for measuring depression. Arch Gen Psychiatry.

[B68] Nemets B, Stahl Z, Belmaker RH (2002). Addition of omega-3 fatty acid to maintenance medication treatment for recurrent unipolar depressive disorder. Am J Psychiatry.

[B69] Su KP, Huang SY, Chiu CC, Shen WW (2003). Omega-3 fatty acids in major depressive disorder. A preliminary double-blind, placebo-controlled trial. Eur Neuropsychopharmacol.

[B70] Zalsman G, Brent DA, Weersing VR (2006). Depressive disorders in childhood and adolescence: an overview: epidemiology, clinical manifestation and risk factors. Child Adolesc Psychiatr Clin N Am.

[B71] Nemets H, Nemets B, Apter A, Bracha Z, Belmaker RH (2006). Omega-3 treatment of childhood depression: a controlled, double-blind pilot study. Am J Psychiatry.

[B72] Poznanski EO, Cook SC, Carroll BJ (1979). A depression rating scale for children. Pediatrics.

[B73] Guy W (1976). ECDEU Assessment Manual for Psychopharmacology – Revised.

[B74] Marangell LB, Martinez JM, Zboyan HA, Kertz B, Kim HF, Puryear LJ (2003). A double-blind, placebo-controlled study of the omega-3 fatty acid docosahexaenoic acid in the treatment of major depression. Am J Psychiatry.

[B75] Silvers KM, Woolley CC, Hamilton FC, Watts PM, Watson RA (2005). Randomised double-blind placebo-controlled trial of fish oil in the treatment of depression. Prostaglandins Leukot Essent Fatty Acids.

[B76] Mitchell PB, Malhi GS (2004). Bipolar depression: phenomenological overview and clinical characteristics. Bipolar Disord.

[B77] Stoll AL, Severus WE, Freeman MP, Rueter S, Zboyan HA, Diamond E, Cress KK, Marangell LB (1999). Omega 3 fatty acids in bipolar disorder: a preliminary double-blind, placebo-controlled trial. Arch Gen Psychiatry.

[B78] Luborsky L (1962). Clinician's judgments of mental health. Arch Gen Psychiatry.

[B79] Young RC, Biggs JT, Ziegler VE, Meyer DA (1978). A rating scale for mania: reliability, validity and sensitivity. Br J Psychiatry.

[B80] Frangou S, Lewis M, McCrone P (2006). Efficacy of ethyl-eicosapentaenoic acid in bipolar depression: randomised double-blind placebo-controlled study. Br J Psychiatry.

[B81] Keck PE, Mintz J, McElroy SL, Freeman MP, Suppes T, Frye MA, Altshuler LL, Kupka R, Nolen WA, Leverich GS, Denicoff KD, Gunze H, Duan N, Post RM (2006). Double-blind, randomized, placebo-controlled trials of ethyl-eicosapenanoate in the treatment of bipolar depression and rapid cycling bipolar disorder. Bio Psychiatry.

[B82] Rush AJ, Giles DE, Schlesser MA, Fulton CL, Weissenburger J, Burns C (1986). The Inventory for Depressive Symptomatology (IDS): preliminary findings. Psychiatry Res.

[B83] Marangell LB, Suppes T, Ketter TA, Dennehy EB, Zboyan H, Kertz B, Nierenberg A, Calabrese J, Wisniewski SR, Sachs G (2006). Omega-3 fatty acids in bipolar disorder: clinical and research considerations. Prostaglandins Leukot Essent Fatty Acids.

[B84] De Vriese SR, Christophe AB, Maes M (2003). Lowered serum n-3 polyunsaturated fatty acid (PUFA) levels predict the occurrence of postpartum depression: further evidence that lowered n-PUFAs are related to major depression. Life Sci.

[B85] Wen SW, Walker M (2004). The use of selective serotonin reuptake inhibitors in pregnancy. J Obstet Gynaecol Can.

[B86] Llorente AM, Jensen CL, Voigt RG, Fraley JK, Berretta MC, Heird WC (2003). Effect of maternal docosahexaenoic acid supplementation on postpartum depression and information processing. Am J Obstet Gynecol.

[B87] Freeman MP, Hibbeln JR, Wisner KL, Brumbach BH, Watchman M, Gelenberg AJ (2006). Randomized dose-ranging pilot trial of omega-3 fatty acids for postpartum depression. Acta Psychiatr Scand.

[B88] Marangell LB, Martinez JM, Zboyan HA, Chong H, Puryear LJ (2004). Omega-3 fatty acids for the prevention of postpartum depression: negative data from a preliminary, open-label pilot study. Depress Anxiety.

[B89] Freeman MP, Hibbeln JR, Wisner KL, Davis JM, Mischoulon D, Peet M, Keck PE, Marangell LB, Richardson AJ, Lake J, Stoll AL (2006). Omega-3 fatty acids: evidence basis for treatment and future research in psychiatry. J Clin Psychiatry.

[B90] Nutt DJ (2006). The role of dopamine and norepinephrine in depression and antidepressant treatment. J Clin Psychiatry.

[B91] Chalon S (2006). Omega-3 fatty acids and monoamine neurotransmission. Prostaglandins Leukot Essent Fatty Acids.

[B92] Kodas E, Galineau S, Bodard S, Vancassel S, Guilloteau D, Besnard J-C, Chalon S (2004). Serotoninergic neurotransmission is affected by n-3 polyunsaturated fatty acids in the rat. J Neurochem.

[B93] Kodas E, Vancassel S, Lejeune B, Guilloteau D, Chalon S (2002). Reversibility of n-3 fatty acid deficiency-induced changes in dopaminergic neurotransmission in rats: critical role of developmental stage. J Lipid Res.

[B94] Zimmer L, Vancassel S, Cantagrel S, Breton P, Delamanche S, Guilloteau D, Durand G, Chalon S (2000). Chronic n-3 polyunsaturated fatty acid deficiency alters dopamine vesicle density in the rat frontal cortex. Neurosci Lett.

[B95] Zimmer L, Hembert S, Durand G, Breton P, Guilloteau D, Besnard J-C, Chalon S (1998). Chronic n-3 polyunstaturated fatty acid diet-deficiency acts on dopamine metabolism in rat frontal cortex: a microdialysis study. Neurosci Lett.

[B96] Levant B, Radel JD, Carlson SE (2004). Decreased brain docosahexaenoic acid during development alters dopamine-related behaviors in adult rats that are differentially affected by dietary remediation. Behavioural Brain Research.

[B97] Zipursky RB, Meyer JH, Verhoeff NP (2007). PET and SPECT imaging in psychiatric disorders. Can J Psychiatry.

[B98] Vial D, Piomelli D (1995). Dopamine D2 receptors potentiate arachidonate release via activation of cytosolic, arachidonic-specific phospholipase A_2_. J Neurochem.

[B99] Piomelli D, Pilon C, Giros B, Sokoloff P, Martres MP, Schwartz JC (1991). Dopamine activation of the arachidonic acid cascade as a basis for D1/D2 receptor synergism. Nature.

[B100] Berg KA, Maayani S, Goldfarb J, Scaramellini C, Leff P, Clarke WP (1998). Effector pathway-dependent relative efficacy at serotonin type 2A and 2C receptors: evidence for agonist-directed trafficking of receptor stimulus. Mol Pharmacol.

[B101] Evans KL, Cropper JD, Berg KA, Clarke WP (2001). Mechanisms of regulation of agonist efficacy at the 5-HT(1A) receptor by phospholipid-derived signaling components. J Pharmacol Exp Ther.

[B102] Horrocks LA, Farooqui AA (2004). Docosahexaenoic acid in the diet: its importance in maintenance and restoration of neural membrane function. Prostaglandins Leukot Essent Fatty Acids.

[B103] Ringbom T, Huss U, Stenholm A, Flock S, Skattebol L, Perera P, Bohlin L (2001). COX-2 inhibitory effects of naturally occurring and modified fatty acids. J Nat Prod.

[B104] Sergeeva M, Strokin M, Reiser G (2005). Regulation of intracellular calcium levels by polyunsaturated fatty acids, arachidonic acid and docosahexaenoic acid, in astrocytes: possible involvement of phospholipase A_2_. Reprod Nutr Dev.

[B105] Berge RK, Masen L, Vaagenes H, Tronstad KJ, Gottlicher M, Rustan AC (1999). In contrast with docosahexaenoic acid, eicosapentaenoic acid and hypolipidaemic derivatives decrease hepatic synthesis and secretion of triacylglycerol by decreased diacylglycerol acyltransferase activity and stimulation of fatty acid oxidation. Biochem J.

[B106] Obajimi O, Black KD, MacDonald DJ, Boyle RM, Glen I, Ross BM (2005). Differential effects of eicosapentaenoic and docosahexaenoic acids upon oxidant-stimulated release and uptake of arachidonic acid in human lymphoma U937 cells. Pharmacol Res.

[B107] Ross BM, Kish SJ (1994). Characterization of lysophospholipid metabolizing enzymes in human brain. J Neurochem.

[B108] Covault J, Pettinati H, Moak D, Mueller T, Kranzler HR (2004). Association of a long-chain fatty acid-CoA ligase 4 gene polymorphism with depression and with enhanced niacin-induced dermal erythema. Am J Med Genet B Neuropsychiatr Genet.

[B109] Horrobin DF, Jenkins K, Bennett CN, Christie WW (2002). Eicosapentaenoic acid and arachidonic acid: collaboration and not antagonism is the key to biological understanding. Prostaglandins Leukot Essent Fatty Acids.

[B110] Colquhoun A, Ramos KL, Schumacher RI (2001). Eicosapentaenoic acid and docosahexaenoic acid effects on tumour mitochondrial metabolism, acyl CoA metabolism and cell proliferation. Cell Biochem Funct.

[B111] Philbrick D-J, Mahadevappa VG, Ackman RG, Holub BJ (1987). Ingestion of fish oil or a derived n-3 fatty acid concentrate containing aicosapentaenoic acid (EPA) affects fatty acid compositions of individual phospholipids of rat brain, sciatic nerve and retina. J Nutr.

[B112] Innis SM, Rioux FM, Auestad N, Ackman RG (1995). Marine and freshwater fish oil varying in arachidonic, eicosapentaenoic and docosahexaenoic acids differ in their effects on organ lipids and fatty acids in growing rats. J Nutr.

[B113] Hawkes JS, James MJ, Cleland LG (1991). Separation and quantification of PGE3 following derivatization with panacyl bromide by high pressure liquid chromatography with fluorometric detection. Prostaglandins.

[B114] Serhan CN, Arita M, Hong S, Gotlinger K (2004). Resolvins, docosatrienes, and neuroprotectins, novel omega-3-derived mediators, and their endogenous aspirin-triggered epimers. Lipids.

[B115] Marcheselli VL, Hong S, Lukiw WJ, Tian XH, Gronert K, Musto A, Hardy M, Gimenez JM, Chiang N, Serhan CN, Bazan NG (2003). Novel docosanoids inhibit brain ischemia-reperfusion-mediated leukocyte infiltration and pro-inflammatory gene expression. J Biol Chem.

[B116] Hong S, Gronert K, Devchand PR, Moussignac RL, Serhan CN (2003). Novel docosatrienes and 17S-resolvins generated from docosahexaenoic acid in murine brain, human blood, and glial cells. Autacoids in anti-inflammation. J Biol Chem.

